# (4*R*,11*R*)-9-(1-hydroxypropan-2-yl)-4,11-diphenyl-1,3,5,7,9-pentaazatri­cyclo[5.3.1.0^4,11^]undecane-2,6-dithione

**DOI:** 10.1107/S160053681102736X

**Published:** 2011-07-16

**Authors:** Meng Wang, Jungang Wang, Jiacheng Xiang

**Affiliations:** a Key Laboratory of Pesticide and Chemical Biology of the Ministry of Education, College of Chemistry, Central China Normal University, Wuhan 430079, People’s Republic of China

## Abstract

The asymmetric unit of the title compound, C_21_H_23_N_5_OS_2_, contains two independent chiral mol­ecules. The two phenyl rings of one mol­ecule form a dihedral angle of 51.95 (7)° and the distance between their centroids is 4.345 (1) Å. In the other mol­ecule, the phenyl rings form a dihedral angle of 58.79 (8)° with a ring centroid–centroid distance of 4.435 (2) Å. An intra­molecular O—H⋯N hydrogen bond occurs in each independent mol­ecule. The crystal packing is stabilized by and inter­molecular N—H⋯O and N—H⋯S hydrogen bonds and C—H⋯S inter­actions.

## Related literature

For crystal engineering studies of similar compounds, see: Deng *et al.* (2010 ▶); Wang & Xi (2009 ▶). For the preparation of the title compound, see: Cao *et al.* (2010 ▶); Li *et al.* (2008 ▶).
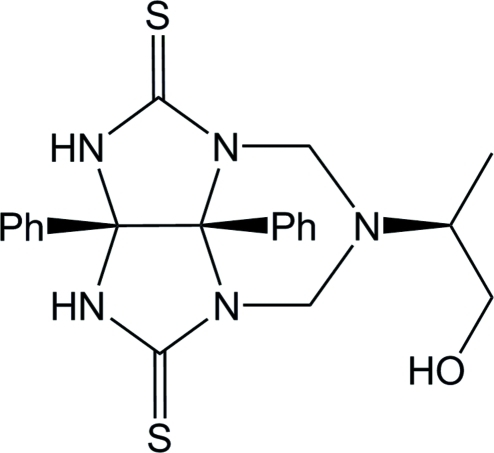


## Experimental

### 

#### Crystal data

C_21_H_23_N_5_OS_2_
*M* *_r_* = 425.58
Monoclinic, 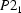

*a* = 9.0030 (9) Å
*b* = 12.6382 (13) Å
*c* = 18.5830 (2) Å
β = 100.169 (2)°
*V* = 2081.2 (4) Å^3^
*Z* = 4
Mo *K*α radiation
μ = 0.28 mm^−1^
*T* = 298 K
0.25 × 0.20 × 0.20 mm

#### Data collection

Bruker SMART 4K CCD area-detector diffractometer
14076 measured reflections
9634 independent reflections
8871 reflections with *I* > 2σ(*I*)
*R*_int_ = 0.020

#### Refinement

*R*[*F* ^2^ > 2σ(*F* ^2^)] = 0.055
*wR*(*F* ^2^) = 0.133
*S* = 1.08
9634 reflections
543 parameters
1 restraint
H atoms treated by a mixture of independent and constrained refinement
Δρ_max_ = 0.39 e Å^−3^
Δρ_min_ = −0.25 e Å^−3^
Absolute structure: Flack (1983 ▶), 4300 Friedel pairs
Flack parameter: 0.04 (6)

### 

Data collection: *SMART* (Bruker, 2001 ▶); cell refinement: *SAINT-Plus* (Bruker, 2001 ▶); data reduction: *SAINT-Plus*; program(s) used to solve structure: *SHELXS97* (Sheldrick, 2008 ▶); program(s) used to refine structure: *SHELXL97* (Sheldrick, 2008 ▶); molecular graphics: *PLATON* (Spek, 2009 ▶); software used to prepare material for publication: *PLATON*, *SHELXL97* and *publCIF* (Westrip, 2010 ▶).

## Supplementary Material

Crystal structure: contains datablock(s) I, global. DOI: 10.1107/S160053681102736X/pk2329sup1.cif

Structure factors: contains datablock(s) I. DOI: 10.1107/S160053681102736X/pk2329Isup2.hkl

Additional supplementary materials:  crystallographic information; 3D view; checkCIF report

## Figures and Tables

**Table 1:** Hydrogen-bond geometry (Å, °)

*D*—H⋯*A*	*D*—H	H⋯*A*	*D*⋯*A*	*D*—H⋯*A*
N10—H10*A*⋯S3^i^	0.76 (4)	2.86 (4)	3.584 (3)	160 (3)
N9—H9*A*⋯S4^ii^	0.85 (4)	2.79 (4)	3.542 (3)	148 (3)
N9—H9*A*⋯O2^iii^	0.85 (4)	2.36 (4)	2.913 (3)	124 (3)
N5—H5*A*⋯S1^iv^	0.79 (4)	2.63 (4)	3.400 (2)	169 (3)
N4—H4*A*⋯O1^iii^	0.83 (3)	2.39 (3)	2.940 (3)	124 (3)
C22—H22*B*⋯S4^v^	0.97	2.85	3.578 (3)	132
C20—H20*B*⋯S1^vi^	0.97	2.85	3.548 (3)	129
O1—H1⋯N1	0.96 (4)	2.04 (3)	2.685 (3)	123 (3)
O2—H2⋯N6	0.97 (4)	1.95 (4)	2.682 (3)	130 (3)

Symmetry codes: (i) 
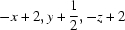
; (ii) 
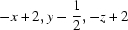
; (iii) 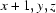; (iv) 
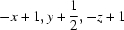
; (v) 
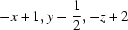
; (vi) 
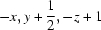
.

**Fig. 1.**
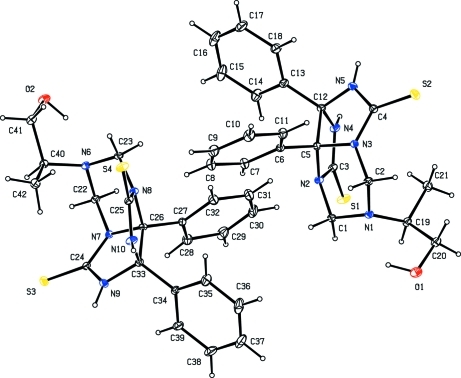
showing the asymmetric unit, with displacement ellipsoids drawn at the 30% probability level.

## supplementary crystallographic information

### Comment

Glycoluril derivatives have been applied in many fields, including explosives and as slow-release fertilizers.  The title compound was synthesized as part of our extensive research on derivatives of glycoluril.  The molecular structure is shown in Fig. 1.  The triazine six-membered ring displays a normal chair conformation.  In the crystal structure, intramolecular O—H···N and intermolecular N—H···O hydrogen bonds as well as weak intermolecular N—H···S hydrogen bonds help to establish the packing.

### Experimental

The title compound was synthesized according to the reported literature (Li *et al.*, 2008). Crystals of the title compound suitable for X-ray diffraction were grown by slow evaporation of a dichloromethane-methanol (4:1) solution of the title compound at 293 K.

### Refinement

All H atoms bound to carbon were placed in geometrically idealized positions and constrained to ride on their parent atoms, with C—H distances in the range 0.93–0.98 Å and U_iso_(H) = 1.2U_eq_(C) or U_iso_(H) = 1.5U_eq_(C_Me_).  All H-atoms bound to nitrogen were found in difference maps and then placed at ideal positions with the N—H = 0.87Å and U_iso_(H) = 1.2U_eq_(N).  Hydroxyl  H-atoms were found in difference maps and refined with U_iso_(H) = 1.5U_eq_(O).

The structure is chiral, and the Flack parameter refines to a satisfactory value of x = 0.04 (6).  Nevertheless, tests using Platon (Spek, 2009) suggest transformation to space group P2_1_/c.  This possibility was tested, but it gave a disordered model that was clearly flawed.

### Crystal data

**Table:**

C_21_H_23_N_5_OS_2_	*F*(000) = 896
*M**_r_* = 425.58	*D*_x_ = 1.358 Mg m^−^^3^
Monoclinic, *P*2_1_	Mo *K*α radiation, λ = 0.71073 Å
Hall symbol: P 2yb	Cell parameters from 4930 reflections
*a* = 9.0030 (9) Å	θ = 0.0–0.0°
*b* = 12.6382 (13) Å	µ = 0.28 mm^−^^1^
*c* = 18.5830 (2) Å	*T* = 298 K
β = 100.169 (2)°	Block, colorless
*V* = 2081.2 (4) Å^3^	0.25 × 0.20 × 0.20 mm
*Z* = 4	

### Data collection

**Table:**

Bruker SMART 4K CCD area-detector diffractometer	8871 reflections with *I* > 2σ(*I*)
Radiation source: fine-focus sealed tube	*R*_int_ = 0.020
graphite	θ_max_ = 28.3°, θ_min_ = 2.0°
φ and ω scans	*h* = −9→11
14076 measured reflections	*k* = −16→16
9634 independent reflections	*l* = −24→22

### Refinement

**Table:**

Refinement on *F*^2^	Secondary atom site location: difference Fourier map
Least-squares matrix: full	Hydrogen site location: inferred from neighbouring sites
*R*[*F*^2^ > 2σ(*F*^2^)] = 0.055	H atoms treated by a mixture of independent and constrained refinement
*wR*(*F*^2^) = 0.133	*w* = 1/[σ^2^(*F*_o_^2^) + (0.0728*P*)^2^ + 0.1223*P*] where *P* = (*F*_o_^2^ + 2*F*_c_^2^)/3
*S* = 1.08	(Δ/σ)_max_ = 0.001
9634 reflections	Δρ_max_ = 0.39 e Å^−^^3^
543 parameters	Δρ_min_ = −0.25 e Å^−^^3^
1 restraint	Absolute structure: Flack (1983), 4300 Friedel pairs
Primary atom site location: structure-invariant direct methods	Flack parameter: 0.04 (6)

### Special details

**Table:**

Geometry. All e.s.d.'s (except the e.s.d. in the dihedral angle between two l.s. planes) are estimated using the full covariance matrix. The cell e.s.d.'s are taken into account individually in the estimation of e.s.d.'s in distances, angles and torsion angles; correlations between e.s.d.'s in cell parameters are only used when they are defined by crystal symmetry. An approximate (isotropic) treatment of cell e.s.d.'s is used for estimating e.s.d.'s involving l.s. planes.
Refinement. Refinement of *F*^2^ against ALL reflections. The weighted *R*-factor *wR* and goodness of fit *S* are based on *F*^2^, conventional *R*-factors *R* are based on *F*, with *F* set to zero for negative *F*^2^. The threshold expression of *F*^2^ > σ(*F*^2^) is used only for calculating *R*-factors(gt) *etc*. and is not relevant to the choice of reflections for refinement.*R*-factors based on *F*^2^ are statistically about twice as large as those based on *F*, and *R*-factors based on ALL data will be even larger.

### Fractional atomic coordinates and isotropic or equivalent isotropic displacement parameters (Å^2^)

**Table:**

	*x*	*y*	*z*	*U*_iso_*/*U*_eq_	
C1	0.0087 (3)	0.7199 (2)	0.40140 (14)	0.0327 (5)	
H1A	−0.0524	0.7447	0.3563	0.039*	
H1B	−0.0158	0.6461	0.4075	0.039*	
C2	0.0195 (3)	0.8891 (2)	0.46139 (15)	0.0333 (6)	
H2A	0.0063	0.9231	0.5067	0.040*	
H2B	−0.0427	0.9263	0.4214	0.040*	
C3	0.2791 (3)	0.6653 (2)	0.43454 (15)	0.0333 (6)	
C4	0.2955 (3)	0.9396 (2)	0.49851 (14)	0.0318 (6)	
C5	0.2239 (3)	0.8369 (2)	0.39335 (13)	0.0279 (5)	
C6	0.1707 (3)	0.8900 (2)	0.31971 (14)	0.0323 (6)	
C7	0.1547 (4)	0.8312 (3)	0.25592 (15)	0.0458 (7)	
H7	0.1756	0.7591	0.2577	0.055*	
C8	0.1069 (4)	0.8815 (3)	0.18885 (18)	0.0597 (10)	
H8	0.0978	0.8426	0.1458	0.072*	
C9	0.0734 (4)	0.9867 (3)	0.18586 (19)	0.0608 (10)	
H9	0.0403	1.0190	0.1409	0.073*	
C10	0.0885 (4)	1.0451 (3)	0.24900 (19)	0.0576 (9)	
H10	0.0653	1.1168	0.2468	0.069*	
C11	0.1385 (4)	0.9968 (3)	0.31635 (16)	0.0449 (7)	
H11	0.1503	1.0367	0.3591	0.054*	
C12	0.4004 (3)	0.8274 (2)	0.42058 (14)	0.0298 (5)	
C13	0.5006 (3)	0.8395 (2)	0.36367 (15)	0.0356 (6)	
C14	0.5248 (4)	0.7536 (3)	0.3207 (2)	0.0571 (9)	
H14	0.4799	0.6889	0.3273	0.069*	
C15	0.6163 (5)	0.7638 (4)	0.2678 (2)	0.0754 (13)	
H15	0.6295	0.7064	0.2382	0.090*	
C16	0.6858 (5)	0.8568 (5)	0.2592 (2)	0.0794 (15)	
H16	0.7484	0.8627	0.2246	0.095*	
C17	0.6640 (4)	0.9408 (4)	0.3010 (2)	0.0708 (12)	
H17	0.7120	1.0045	0.2948	0.085*	
C18	0.5709 (4)	0.9339 (3)	0.35345 (18)	0.0513 (8)	
H18	0.5563	0.9927	0.3814	0.062*	
C19	−0.0175 (3)	0.7270 (2)	0.53353 (14)	0.0348 (5)	
H19	−0.0373	0.6516	0.5240	0.042*	
C20	−0.1461 (3)	0.7702 (3)	0.56985 (17)	0.0453 (7)	
H20A	−0.1593	0.7246	0.6102	0.054*	
H20B	−0.1195	0.8403	0.5893	0.054*	
C21	0.1319 (3)	0.7357 (3)	0.58612 (16)	0.0485 (8)	
H21A	0.2112	0.7071	0.5635	0.073*	
H21B	0.1262	0.6968	0.6299	0.073*	
H21C	0.1528	0.8087	0.5981	0.073*	
C22	0.5347 (3)	0.8098 (2)	0.95706 (14)	0.0305 (5)	
H22A	0.4753	0.7825	0.9122	0.037*	
H22B	0.5229	0.7616	0.9963	0.037*	
C23	0.5052 (3)	0.9915 (2)	0.91961 (14)	0.0307 (5)	
H23A	0.4790	1.0609	0.9358	0.037*	
H23B	0.4407	0.9771	0.8731	0.037*	
C24	0.8133 (3)	0.7917 (2)	1.00265 (13)	0.0301 (5)	
C25	0.7688 (3)	1.0667 (2)	0.93261 (14)	0.0322 (6)	
C26	0.7301 (3)	0.89034 (19)	0.89609 (13)	0.0267 (5)	
C27	0.6763 (3)	0.8523 (2)	0.81857 (14)	0.0321 (6)	
C28	0.6589 (4)	0.7455 (3)	0.80296 (17)	0.0485 (8)	
H28	0.6844	0.6958	0.8400	0.058*	
C29	0.6036 (4)	0.7124 (4)	0.7324 (2)	0.0682 (11)	
H29	0.5889	0.6406	0.7226	0.082*	
C30	0.5708 (4)	0.7836 (5)	0.67732 (19)	0.0745 (14)	
H30	0.5334	0.7606	0.6300	0.089*	
C31	0.5924 (4)	0.8889 (5)	0.6912 (2)	0.0729 (13)	
H31	0.5733	0.9373	0.6529	0.088*	
C32	0.6426 (4)	0.9244 (3)	0.76170 (17)	0.0530 (8)	
H32	0.6537	0.9966	0.7710	0.064*	
C33	0.9044 (3)	0.9102 (2)	0.92241 (13)	0.0291 (5)	
C34	1.0088 (3)	0.8872 (2)	0.86789 (14)	0.0327 (6)	
C35	1.0333 (4)	0.9641 (3)	0.81817 (19)	0.0567 (9)	
H35	0.9854	1.0294	0.8175	0.068*	
C36	1.1301 (5)	0.9430 (5)	0.7692 (2)	0.0803 (14)	
H36	1.1459	0.9946	0.7356	0.096*	
C37	1.2020 (4)	0.8481 (5)	0.7696 (2)	0.0774 (15)	
H37	1.2673	0.8351	0.7370	0.093*	
C38	1.1775 (4)	0.7728 (4)	0.8180 (2)	0.0696 (12)	
H38	1.2271	0.7081	0.8187	0.084*	
C39	1.0797 (3)	0.7906 (3)	0.86668 (17)	0.0478 (8)	
H39	1.0619	0.7372	0.8986	0.057*	
C40	0.4939 (3)	0.9423 (2)	1.05056 (14)	0.0325 (5)	
H40	0.4941	0.8765	1.0785	0.039*	
C41	0.3536 (3)	1.0042 (2)	1.06007 (16)	0.0413 (6)	
H41A	0.3474	1.0079	1.1116	0.050*	
H41B	0.3609	1.0759	1.0424	0.050*	
C42	0.6351 (3)	1.0042 (3)	1.08372 (16)	0.0441 (7)	
H42A	0.7228	0.9613	1.0830	0.066*	
H42B	0.6297	1.0229	1.1333	0.066*	
H42C	0.6417	1.0673	1.0557	0.066*	
N1	−0.0297 (2)	0.78024 (17)	0.46192 (12)	0.0315 (5)	
N2	0.1697 (2)	0.72850 (18)	0.39543 (12)	0.0309 (5)	
N3	0.1782 (2)	0.89701 (18)	0.45329 (11)	0.0290 (4)	
N4	0.4072 (2)	0.7193 (2)	0.45007 (13)	0.0364 (5)	
H4A	0.486 (4)	0.688 (3)	0.4681 (16)	0.044*	
N5	0.4231 (3)	0.9080 (2)	0.47628 (13)	0.0381 (5)	
H5A	0.503 (4)	0.930 (3)	0.4934 (18)	0.046*	
N6	0.4774 (2)	0.91310 (17)	0.97243 (11)	0.0294 (4)	
N7	0.6935 (2)	0.81281 (17)	0.94950 (11)	0.0261 (4)	
N8	0.6638 (2)	0.99257 (17)	0.90927 (11)	0.0289 (4)	
N9	0.9352 (3)	0.8419 (2)	0.98643 (13)	0.0365 (5)	
H9A	1.022 (4)	0.832 (3)	1.0115 (17)	0.044*	
N10	0.9063 (3)	1.0212 (2)	0.94075 (14)	0.0400 (6)	
H10A	0.979 (4)	1.054 (3)	0.9465 (17)	0.048*	
O1	−0.2835 (3)	0.7757 (2)	0.51903 (15)	0.0607 (7)	
H1	−0.251 (5)	0.777 (4)	0.472 (2)	0.091*	
O2	0.2204 (2)	0.9554 (2)	1.02108 (13)	0.0496 (6)	
H2	0.268 (4)	0.916 (4)	0.986 (2)	0.074*	
S1	0.25277 (9)	0.53965 (6)	0.45623 (5)	0.0503 (2)	
S2	0.28553 (9)	1.02021 (7)	0.56882 (4)	0.0469 (2)	
S3	0.80953 (8)	0.71417 (6)	1.07516 (4)	0.04121 (18)	
S4	0.73491 (9)	1.19461 (6)	0.94594 (5)	0.0468 (2)	

### Atomic displacement parameters (Å^2^)

**Table:**

	*U*^11^	*U*^22^	*U*^33^	*U*^12^	*U*^13^	*U*^23^
C1	0.0237 (11)	0.0382 (14)	0.0364 (12)	−0.0003 (10)	0.0058 (9)	−0.0016 (11)
C2	0.0213 (11)	0.0389 (15)	0.0402 (14)	0.0049 (10)	0.0067 (10)	0.0013 (11)
C3	0.0262 (13)	0.0354 (15)	0.0410 (14)	0.0021 (10)	0.0132 (11)	0.0025 (11)
C4	0.0285 (13)	0.0339 (14)	0.0320 (13)	0.0017 (11)	0.0023 (10)	0.0015 (11)
C5	0.0219 (12)	0.0319 (13)	0.0299 (12)	0.0027 (10)	0.0050 (9)	−0.0006 (10)
C6	0.0244 (12)	0.0401 (15)	0.0324 (13)	−0.0002 (11)	0.0046 (10)	0.0038 (11)
C7	0.0526 (19)	0.0477 (18)	0.0370 (15)	0.0008 (14)	0.0069 (13)	−0.0048 (13)
C8	0.064 (2)	0.081 (3)	0.0331 (16)	−0.009 (2)	0.0039 (14)	−0.0026 (16)
C9	0.057 (2)	0.078 (3)	0.0445 (18)	−0.0003 (18)	−0.0005 (15)	0.0271 (18)
C10	0.063 (2)	0.053 (2)	0.057 (2)	0.0086 (17)	0.0097 (16)	0.0232 (16)
C11	0.0550 (19)	0.0402 (16)	0.0396 (15)	0.0030 (14)	0.0084 (13)	0.0057 (13)
C12	0.0220 (12)	0.0331 (14)	0.0345 (13)	0.0007 (10)	0.0055 (10)	0.0016 (11)
C13	0.0243 (13)	0.0454 (16)	0.0373 (14)	0.0052 (11)	0.0060 (10)	0.0075 (12)
C14	0.062 (2)	0.053 (2)	0.062 (2)	0.0128 (17)	0.0286 (17)	0.0003 (16)
C15	0.086 (3)	0.090 (3)	0.060 (2)	0.037 (3)	0.040 (2)	0.011 (2)
C16	0.059 (2)	0.123 (4)	0.066 (2)	0.031 (3)	0.037 (2)	0.033 (3)
C17	0.056 (2)	0.095 (3)	0.065 (2)	−0.014 (2)	0.0196 (18)	0.032 (2)
C18	0.0465 (18)	0.058 (2)	0.0504 (18)	−0.0086 (16)	0.0096 (14)	0.0089 (16)
C19	0.0312 (13)	0.0379 (14)	0.0365 (13)	0.0016 (11)	0.0089 (10)	0.0043 (11)
C20	0.0392 (16)	0.0513 (18)	0.0495 (17)	0.0029 (13)	0.0195 (13)	0.0062 (14)
C21	0.0392 (16)	0.067 (2)	0.0381 (15)	0.0068 (15)	0.0028 (12)	0.0070 (15)
C22	0.0243 (12)	0.0340 (13)	0.0342 (12)	−0.0037 (10)	0.0074 (10)	−0.0021 (11)
C23	0.0208 (11)	0.0350 (14)	0.0365 (13)	0.0016 (10)	0.0059 (10)	−0.0015 (11)
C24	0.0264 (12)	0.0338 (14)	0.0308 (12)	0.0015 (10)	0.0071 (10)	−0.0016 (10)
C25	0.0328 (13)	0.0297 (14)	0.0371 (14)	−0.0020 (10)	0.0142 (11)	−0.0020 (11)
C26	0.0236 (11)	0.0234 (12)	0.0336 (12)	0.0012 (9)	0.0061 (9)	0.0018 (10)
C27	0.0231 (12)	0.0461 (16)	0.0268 (12)	0.0005 (11)	0.0038 (9)	−0.0005 (11)
C28	0.0526 (19)	0.0499 (19)	0.0420 (16)	−0.0008 (15)	0.0052 (13)	−0.0103 (13)
C29	0.060 (2)	0.088 (3)	0.057 (2)	−0.010 (2)	0.0114 (17)	−0.035 (2)
C30	0.045 (2)	0.143 (5)	0.0352 (17)	−0.002 (2)	0.0056 (14)	−0.027 (2)
C31	0.053 (2)	0.125 (4)	0.0385 (18)	0.014 (2)	0.0035 (15)	0.019 (2)
C32	0.0459 (17)	0.070 (2)	0.0423 (16)	0.0030 (17)	0.0054 (13)	0.0070 (16)
C33	0.0222 (11)	0.0346 (14)	0.0314 (12)	−0.0043 (10)	0.0073 (9)	−0.0030 (11)
C34	0.0208 (12)	0.0446 (16)	0.0340 (13)	−0.0043 (11)	0.0082 (10)	−0.0060 (11)
C35	0.058 (2)	0.063 (2)	0.055 (2)	−0.0059 (17)	0.0263 (16)	0.0022 (17)
C36	0.075 (3)	0.119 (4)	0.055 (2)	−0.030 (3)	0.034 (2)	0.002 (2)
C37	0.045 (2)	0.137 (5)	0.055 (2)	−0.012 (2)	0.0228 (17)	−0.041 (3)
C38	0.044 (2)	0.098 (3)	0.066 (2)	0.018 (2)	0.0060 (17)	−0.041 (2)
C39	0.0399 (16)	0.055 (2)	0.0485 (17)	0.0098 (14)	0.0079 (13)	−0.0149 (15)
C40	0.0260 (12)	0.0378 (14)	0.0346 (13)	−0.0026 (10)	0.0077 (10)	0.0012 (11)
C41	0.0382 (15)	0.0424 (16)	0.0463 (15)	−0.0026 (12)	0.0156 (12)	−0.0068 (13)
C42	0.0358 (15)	0.0544 (18)	0.0406 (14)	−0.0059 (14)	0.0022 (12)	−0.0086 (14)
N1	0.0242 (10)	0.0359 (12)	0.0355 (11)	0.0008 (9)	0.0081 (8)	0.0002 (9)
N2	0.0248 (10)	0.0316 (11)	0.0366 (11)	0.0006 (9)	0.0067 (8)	−0.0003 (9)
N3	0.0234 (10)	0.0341 (11)	0.0291 (10)	0.0007 (9)	0.0035 (8)	−0.0019 (9)
N4	0.0244 (10)	0.0406 (14)	0.0441 (12)	0.0041 (10)	0.0062 (9)	0.0117 (11)
N5	0.0239 (11)	0.0490 (15)	0.0401 (12)	−0.0041 (10)	0.0022 (9)	−0.0090 (11)
N6	0.0231 (10)	0.0304 (11)	0.0359 (11)	−0.0018 (8)	0.0081 (8)	−0.0020 (9)
N7	0.0219 (10)	0.0259 (10)	0.0313 (10)	−0.0031 (8)	0.0075 (8)	−0.0006 (8)
N8	0.0227 (10)	0.0277 (11)	0.0376 (11)	−0.0007 (8)	0.0087 (8)	0.0017 (9)
N9	0.0201 (11)	0.0537 (15)	0.0346 (11)	−0.0020 (10)	0.0017 (9)	0.0090 (10)
N10	0.0240 (11)	0.0359 (14)	0.0611 (15)	−0.0073 (10)	0.0104 (10)	−0.0137 (12)
O1	0.0304 (12)	0.085 (2)	0.0696 (16)	0.0005 (12)	0.0163 (11)	−0.0061 (14)
O2	0.0270 (10)	0.0561 (14)	0.0691 (15)	−0.0041 (9)	0.0175 (9)	−0.0051 (12)
S1	0.0360 (4)	0.0341 (4)	0.0830 (6)	0.0057 (3)	0.0172 (4)	0.0177 (4)
S2	0.0392 (4)	0.0556 (5)	0.0442 (4)	0.0031 (3)	0.0024 (3)	−0.0186 (4)
S3	0.0374 (4)	0.0496 (4)	0.0371 (3)	0.0019 (3)	0.0078 (3)	0.0118 (3)
S4	0.0386 (4)	0.0296 (4)	0.0768 (6)	−0.0032 (3)	0.0232 (4)	−0.0088 (4)

### Geometric parameters (Å, °)

**Table:**

C1—N1	1.450 (3)	C22—H22B	0.9700
C1—N2	1.477 (3)	C23—N6	1.447 (3)
C1—H1A	0.9700	C23—N8	1.474 (3)
C1—H1B	0.9700	C23—H23A	0.9700
C2—N1	1.447 (3)	C23—H23B	0.9700
C2—N3	1.466 (3)	C24—N9	1.347 (3)
C2—H2A	0.9700	C24—N7	1.354 (3)
C2—H2B	0.9700	C24—S3	1.671 (3)
C3—N4	1.327 (3)	C25—N8	1.347 (3)
C3—N2	1.373 (3)	C25—N10	1.350 (4)
C3—S1	1.666 (3)	C25—S4	1.671 (3)
C4—N3	1.341 (3)	C26—N8	1.462 (3)
C4—N5	1.348 (3)	C26—N7	1.473 (3)
C4—S2	1.672 (3)	C26—C27	1.515 (3)
C5—N2	1.458 (3)	C26—C33	1.579 (3)
C5—N3	1.466 (3)	C27—C28	1.384 (4)
C5—C6	1.523 (3)	C27—C32	1.388 (4)
C5—C12	1.584 (3)	C28—C29	1.383 (4)
C6—C11	1.381 (4)	C28—H28	0.9300
C6—C7	1.385 (4)	C29—C30	1.356 (7)
C7—C8	1.397 (5)	C29—H29	0.9300
C7—H7	0.9300	C30—C31	1.363 (7)
C8—C9	1.363 (6)	C30—H30	0.9300
C8—H8	0.9300	C31—C32	1.383 (5)
C9—C10	1.372 (5)	C31—H31	0.9300
C9—H9	0.9300	C32—H32	0.9300
C10—C11	1.393 (4)	C33—N10	1.443 (4)
C10—H10	0.9300	C33—N9	1.457 (3)
C11—H11	0.9300	C33—C34	1.527 (3)
C12—N5	1.442 (4)	C34—C39	1.380 (4)
C12—N4	1.469 (4)	C34—C35	1.385 (4)
C12—C13	1.514 (4)	C35—C36	1.393 (5)
C13—C18	1.379 (4)	C35—H35	0.9300
C13—C14	1.387 (5)	C36—C37	1.363 (7)
C14—C15	1.397 (5)	C36—H36	0.9300
C14—H14	0.9300	C37—C38	1.354 (7)
C15—C16	1.354 (7)	C37—H37	0.9300
C15—H15	0.9300	C38—C39	1.387 (5)
C16—C17	1.351 (7)	C38—H38	0.9300
C16—H16	0.9300	C39—H39	0.9300
C17—C18	1.396 (5)	C40—N6	1.480 (3)
C17—H17	0.9300	C40—C41	1.523 (4)
C18—H18	0.9300	C40—C42	1.527 (4)
C19—N1	1.478 (3)	C40—H40	0.9800
C19—C21	1.521 (4)	C41—O2	1.427 (4)
C19—C20	1.539 (4)	C41—H41A	0.9700
C19—H19	0.9800	C41—H41B	0.9700
C20—O1	1.420 (4)	C42—H42A	0.9600
C20—H20A	0.9700	C42—H42B	0.9600
C20—H20B	0.9700	C42—H42C	0.9600
C21—H21A	0.9600	N4—H4A	0.83 (3)
C21—H21B	0.9600	N5—H5A	0.79 (4)
C21—H21C	0.9600	N9—H9A	0.85 (4)
C22—N6	1.451 (3)	N10—H10A	0.76 (4)
C22—N7	1.461 (3)	O1—H1	0.96 (4)
C22—H22A	0.9700	O2—H2	0.97 (4)
			
N1—C1—N2	113.0 (2)	N8—C25—S4	125.8 (2)
N1—C1—H1A	109.0	N10—C25—S4	125.7 (2)
N2—C1—H1A	109.0	N8—C26—N7	109.17 (19)
N1—C1—H1B	109.0	N8—C26—C27	111.6 (2)
N2—C1—H1B	109.0	N7—C26—C27	111.0 (2)
H1A—C1—H1B	107.8	N8—C26—C33	102.74 (19)
N1—C2—N3	111.8 (2)	N7—C26—C33	103.04 (19)
N1—C2—H2A	109.3	C27—C26—C33	118.6 (2)
N3—C2—H2A	109.3	C28—C27—C32	118.6 (3)
N1—C2—H2B	109.3	C28—C27—C26	120.9 (2)
N3—C2—H2B	109.3	C32—C27—C26	120.4 (3)
H2A—C2—H2B	107.9	C29—C28—C27	120.1 (3)
N4—C3—N2	109.3 (2)	C29—C28—H28	119.9
N4—C3—S1	126.4 (2)	C27—C28—H28	119.9
N2—C3—S1	124.3 (2)	C30—C29—C28	120.6 (4)
N3—C4—N5	108.0 (2)	C30—C29—H29	119.7
N3—C4—S2	126.1 (2)	C28—C29—H29	119.7
N5—C4—S2	125.9 (2)	C29—C30—C31	120.1 (3)
N2—C5—N3	109.1 (2)	C29—C30—H30	120.0
N2—C5—C6	112.6 (2)	C31—C30—H30	120.0
N3—C5—C6	111.6 (2)	C30—C31—C32	120.5 (4)
N2—C5—C12	104.0 (2)	C30—C31—H31	119.7
N3—C5—C12	101.38 (19)	C32—C31—H31	119.7
C6—C5—C12	117.3 (2)	C31—C32—C27	120.0 (4)
C11—C6—C7	119.8 (3)	C31—C32—H32	120.0
C11—C6—C5	120.1 (3)	C27—C32—H32	120.0
C7—C6—C5	120.1 (3)	N10—C33—N9	113.0 (2)
C6—C7—C8	119.3 (3)	N10—C33—C34	111.0 (2)
C6—C7—H7	120.4	N9—C33—C34	112.3 (2)
C8—C7—H7	120.4	N10—C33—C26	101.4 (2)
C9—C8—C7	120.7 (3)	N9—C33—C26	101.19 (19)
C9—C8—H8	119.6	C34—C33—C26	117.3 (2)
C7—C8—H8	119.6	C39—C34—C35	118.8 (3)
C8—C9—C10	120.1 (3)	C39—C34—C33	121.3 (3)
C8—C9—H9	119.9	C35—C34—C33	119.9 (3)
C10—C9—H9	119.9	C34—C35—C36	119.6 (4)
C9—C10—C11	120.0 (4)	C34—C35—H35	120.2
C9—C10—H10	120.0	C36—C35—H35	120.2
C11—C10—H10	120.0	C37—C36—C35	121.1 (4)
C6—C11—C10	120.0 (3)	C37—C36—H36	119.5
C6—C11—H11	120.0	C35—C36—H36	119.5
C10—C11—H11	120.0	C38—C37—C36	119.3 (3)
N5—C12—N4	113.5 (2)	C38—C37—H37	120.3
N5—C12—C13	113.8 (2)	C36—C37—H37	120.3
N4—C12—C13	111.4 (2)	C37—C38—C39	121.0 (4)
N5—C12—C5	100.5 (2)	C37—C38—H38	119.5
N4—C12—C5	99.4 (2)	C39—C38—H38	119.5
C13—C12—C5	117.2 (2)	C34—C39—C38	120.2 (4)
C18—C13—C14	118.4 (3)	C34—C39—H39	119.9
C18—C13—C12	121.9 (3)	C38—C39—H39	119.9
C14—C13—C12	119.7 (3)	N6—C40—C41	107.5 (2)
C13—C14—C15	120.4 (4)	N6—C40—C42	117.1 (2)
C13—C14—H14	119.8	C41—C40—C42	109.8 (2)
C15—C14—H14	119.8	N6—C40—H40	107.4
C16—C15—C14	120.3 (4)	C41—C40—H40	107.4
C16—C15—H15	119.8	C42—C40—H40	107.4
C14—C15—H15	119.8	O2—C41—C40	111.0 (2)
C17—C16—C15	119.9 (4)	O2—C41—H41A	109.4
C17—C16—H16	120.1	C40—C41—H41A	109.4
C15—C16—H16	120.1	O2—C41—H41B	109.4
C16—C17—C18	121.2 (4)	C40—C41—H41B	109.4
C16—C17—H17	119.4	H41A—C41—H41B	108.0
C18—C17—H17	119.4	C40—C42—H42A	109.5
C13—C18—C17	119.9 (4)	C40—C42—H42B	109.5
C13—C18—H18	120.1	H42A—C42—H42B	109.5
C17—C18—H18	120.1	C40—C42—H42C	109.5
N1—C19—C21	117.5 (2)	H42A—C42—H42C	109.5
N1—C19—C20	106.9 (2)	H42B—C42—H42C	109.5
C21—C19—C20	109.9 (2)	C2—N1—C1	112.3 (2)
N1—C19—H19	107.3	C2—N1—C19	117.7 (2)
C21—C19—H19	107.3	C1—N1—C19	118.0 (2)
C20—C19—H19	107.3	C3—N2—C5	110.4 (2)
O1—C20—C19	111.1 (2)	C3—N2—C1	122.7 (2)
O1—C20—H20A	109.4	C5—N2—C1	114.1 (2)
C19—C20—H20A	109.4	C4—N3—C2	129.6 (2)
O1—C20—H20B	109.4	C4—N3—C5	112.8 (2)
C19—C20—H20B	109.4	C2—N3—C5	116.9 (2)
H20A—C20—H20B	108.0	C3—N4—C12	114.8 (2)
C19—C21—H21A	109.5	C3—N4—H4A	120 (2)
C19—C21—H21B	109.5	C12—N4—H4A	125 (2)
H21A—C21—H21B	109.5	C4—N5—C12	114.1 (2)
C19—C21—H21C	109.5	C4—N5—H5A	123 (3)
H21A—C21—H21C	109.5	C12—N5—H5A	123 (3)
H21B—C21—H21C	109.5	C23—N6—C22	111.9 (2)
N6—C22—N7	112.5 (2)	C23—N6—C40	119.7 (2)
N6—C22—H22A	109.1	C22—N6—C40	116.1 (2)
N7—C22—H22A	109.1	C24—N7—C22	126.3 (2)
N6—C22—H22B	109.1	C24—N7—C26	112.2 (2)
N7—C22—H22B	109.1	C22—N7—C26	114.94 (19)
H22A—C22—H22B	107.8	C25—N8—C26	112.6 (2)
N6—C23—N8	112.5 (2)	C25—N8—C23	127.2 (2)
N6—C23—H23A	109.1	C26—N8—C23	116.6 (2)
N8—C23—H23A	109.1	C24—N9—C33	114.6 (2)
N6—C23—H23B	109.1	C24—N9—H9A	121 (2)
N8—C23—H23B	109.1	C33—N9—H9A	124 (2)
H23A—C23—H23B	107.8	C25—N10—C33	114.5 (2)
N9—C24—N7	108.6 (2)	C25—N10—H10A	122 (3)
N9—C24—S3	126.1 (2)	C33—N10—H10A	122 (3)
N7—C24—S3	125.2 (2)	C20—O1—H1	103 (3)
N8—C25—N10	108.5 (2)	C41—O2—H2	97 (2)
			
N2—C5—C6—C11	145.7 (3)	N2—C1—N1—C19	−90.0 (3)
N3—C5—C6—C11	22.7 (4)	C21—C19—N1—C2	−48.8 (3)
C12—C5—C6—C11	−93.7 (3)	C20—C19—N1—C2	75.3 (3)
N2—C5—C6—C7	−34.4 (3)	C21—C19—N1—C1	91.1 (3)
N3—C5—C6—C7	−157.5 (2)	C20—C19—N1—C1	−144.8 (2)
C12—C5—C6—C7	86.2 (3)	N4—C3—N2—C5	−8.3 (3)
C11—C6—C7—C8	0.3 (5)	S1—C3—N2—C5	173.3 (2)
C5—C6—C7—C8	−179.5 (3)	N4—C3—N2—C1	−147.4 (2)
C6—C7—C8—C9	−1.2 (6)	S1—C3—N2—C1	34.1 (4)
C7—C8—C9—C10	0.9 (6)	N3—C5—N2—C3	−93.7 (2)
C8—C9—C10—C11	0.2 (6)	C6—C5—N2—C3	141.8 (2)
C7—C6—C11—C10	0.8 (5)	C12—C5—N2—C3	13.8 (3)
C5—C6—C11—C10	−179.4 (3)	N3—C5—N2—C1	49.2 (3)
C9—C10—C11—C6	−1.0 (5)	C6—C5—N2—C1	−75.3 (3)
N2—C5—C12—N5	−129.6 (2)	C12—C5—N2—C1	156.73 (19)
N3—C5—C12—N5	−16.4 (2)	N1—C1—N2—C3	84.9 (3)
C6—C5—C12—N5	105.4 (2)	N1—C1—N2—C5	−52.9 (3)
N2—C5—C12—N4	−13.3 (2)	N5—C4—N3—C2	166.6 (3)
N3—C5—C12—N4	99.8 (2)	S2—C4—N3—C2	−14.8 (4)
C6—C5—C12—N4	−138.4 (2)	N5—C4—N3—C5	−3.4 (3)
N2—C5—C12—C13	106.7 (3)	S2—C4—N3—C5	175.1 (2)
N3—C5—C12—C13	−140.2 (2)	N1—C2—N3—C4	−119.3 (3)
C6—C5—C12—C13	−18.4 (4)	N1—C2—N3—C5	50.4 (3)
N5—C12—C13—C18	−18.6 (4)	N2—C5—N3—C4	122.2 (2)
N4—C12—C13—C18	−148.3 (3)	C6—C5—N3—C4	−112.8 (2)
C5—C12—C13—C18	98.2 (3)	C12—C5—N3—C4	13.0 (3)
N5—C12—C13—C14	160.7 (3)	N2—C5—N3—C2	−49.2 (3)
N4—C12—C13—C14	30.9 (4)	C6—C5—N3—C2	75.9 (3)
C5—C12—C13—C14	−82.6 (3)	C12—C5—N3—C2	−158.4 (2)
C18—C13—C14—C15	−1.1 (5)	N2—C3—N4—C12	−1.8 (3)
C12—C13—C14—C15	179.7 (3)	S1—C3—N4—C12	176.6 (2)
C13—C14—C15—C16	1.9 (6)	N5—C12—N4—C3	115.7 (3)
C14—C15—C16—C17	−1.4 (7)	C13—C12—N4—C3	−114.4 (3)
C15—C16—C17—C18	0.2 (7)	C5—C12—N4—C3	9.8 (3)
C14—C13—C18—C17	−0.2 (5)	N3—C4—N5—C12	−9.4 (3)
C12—C13—C18—C17	179.1 (3)	S2—C4—N5—C12	172.0 (2)
C16—C17—C18—C13	0.7 (6)	N4—C12—N5—C4	−88.6 (3)
N1—C19—C20—O1	43.9 (3)	C13—C12—N5—C4	142.7 (2)
C21—C19—C20—O1	172.5 (3)	C5—C12—N5—C4	16.6 (3)
N8—C26—C27—C28	−147.6 (3)	N8—C23—N6—C22	−50.4 (3)
N7—C26—C27—C28	−25.7 (3)	N8—C23—N6—C40	90.3 (3)
C33—C26—C27—C28	93.3 (3)	N7—C22—N6—C23	53.0 (3)
N8—C26—C27—C32	32.0 (3)	N7—C22—N6—C40	−89.2 (2)
N7—C26—C27—C32	154.0 (2)	C41—C40—N6—C23	77.8 (3)
C33—C26—C27—C32	−87.0 (3)	C42—C40—N6—C23	−46.3 (3)
C32—C27—C28—C29	−2.4 (5)	C41—C40—N6—C22	−143.0 (2)
C26—C27—C28—C29	177.3 (3)	C42—C40—N6—C22	92.9 (3)
C27—C28—C29—C30	2.2 (5)	N9—C24—N7—C22	−155.4 (2)
C28—C29—C30—C31	0.2 (6)	S3—C24—N7—C22	25.8 (4)
C29—C30—C31—C32	−2.4 (6)	N9—C24—N7—C26	−5.6 (3)
C30—C31—C32—C27	2.2 (6)	S3—C24—N7—C26	175.58 (19)
C28—C27—C32—C31	0.2 (5)	N6—C22—N7—C24	95.6 (3)
C26—C27—C32—C31	−179.5 (3)	N6—C22—N7—C26	−53.5 (3)
N8—C26—C33—N10	−3.8 (2)	N8—C26—N7—C24	−104.8 (2)
N7—C26—C33—N10	−117.3 (2)	C27—C26—N7—C24	131.8 (2)
C27—C26—C33—N10	119.7 (2)	C33—C26—N7—C24	3.8 (3)
N8—C26—C33—N9	112.7 (2)	N8—C26—N7—C22	48.6 (3)
N7—C26—C33—N9	−0.8 (2)	C27—C26—N7—C22	−74.8 (3)
C27—C26—C33—N9	−123.8 (2)	C33—C26—N7—C22	157.26 (19)
N8—C26—C33—C34	−124.9 (2)	N10—C25—N8—C26	−3.4 (3)
N7—C26—C33—C34	121.7 (2)	S4—C25—N8—C26	174.25 (19)
C27—C26—C33—C34	−1.3 (3)	N10—C25—N8—C23	154.3 (2)
N10—C33—C34—C39	150.1 (3)	S4—C25—N8—C23	−28.0 (4)
N9—C33—C34—C39	22.6 (4)	N7—C26—N8—C25	113.5 (2)
C26—C33—C34—C39	−94.0 (3)	C27—C26—N8—C25	−123.5 (2)
N10—C33—C34—C35	−30.1 (4)	C33—C26—N8—C25	4.6 (3)
N9—C33—C34—C35	−157.7 (3)	N7—C26—N8—C23	−46.8 (3)
C26—C33—C34—C35	85.7 (3)	C27—C26—N8—C23	76.2 (3)
C39—C34—C35—C36	−1.0 (5)	C33—C26—N8—C23	−155.7 (2)
C33—C34—C35—C36	179.2 (3)	N6—C23—N8—C25	−107.6 (3)
C34—C35—C36—C37	−0.4 (6)	N6—C23—N8—C26	49.4 (3)
C35—C36—C37—C38	0.7 (7)	N7—C24—N9—C33	5.1 (3)
C36—C37—C38—C39	0.5 (6)	S3—C24—N9—C33	−176.0 (2)
C35—C34—C39—C38	2.2 (5)	N10—C33—N9—C24	105.1 (3)
C33—C34—C39—C38	−178.1 (3)	C34—C33—N9—C24	−128.4 (3)
C37—C38—C39—C34	−2.0 (5)	C26—C33—N9—C24	−2.5 (3)
N6—C40—C41—O2	43.7 (3)	N8—C25—N10—C33	0.5 (3)
C42—C40—C41—O2	172.1 (2)	S4—C25—N10—C33	−177.2 (2)
N3—C2—N1—C1	−49.8 (3)	N9—C33—N10—C25	−105.3 (3)
N3—C2—N1—C19	92.3 (3)	C34—C33—N10—C25	127.6 (2)
N2—C1—N1—C2	52.0 (3)	C26—C33—N10—C25	2.2 (3)

### Hydrogen-bond geometry (Å, °)

**Table:**

*D*—H···*A*	*D*—H	H···*A*	*D*···*A*	*D*—H···*A*
N10—H10A···S3^i^	0.76 (4)	2.86 (4)	3.584 (3)	160 (3)
N9—H9A···S4^ii^	0.85 (4)	2.79 (4)	3.542 (3)	148 (3)
N9—H9A···O2^iii^	0.85 (4)	2.36 (4)	2.913 (3)	124 (3)
N5—H5A···S1^iv^	0.79 (4)	2.63 (4)	3.400 (2)	169 (3)
N4—H4A···O1^iii^	0.83 (3)	2.39 (3)	2.940 (3)	124 (3)
C22—H22B···S4^v^	0.97	2.85	3.578 (3)	132
C20—H20B···S1^vi^	0.97	2.85	3.548 (3)	129
O1—H1···N1	0.96 (4)	2.04 (3)	2.685 (3)	123 (3)
O2—H2···N6	0.97 (4)	1.95 (4)	2.682 (3)	130 (3)

Symmetry codes: (i) −*x*+2, *y*+1/2, −*z*+2; (ii) −*x*+2, *y*−1/2, −*z*+2; (iii) *x*+1, *y*, *z*; (iv) −*x*+1, *y*+1/2, −*z*+1; (v) −*x*+1, *y*−1/2, −*z*+2; (vi) −*x*, *y*+1/2, −*z*+1.
